# Special Issue: Biomimetic Organic–Inorganic Composites

**DOI:** 10.3390/ma15093074

**Published:** 2022-04-23

**Authors:** Maja Dutour Sikirić

**Affiliations:** Laboratory for Biocolloids and Surface Chemistry, Division of Physical Chemistry, Ruđer Bošković Institute, Bijenička c. 54, 10000 Zagreb, Croatia; sikiric@irb.hr

Throughout history, the welfare and prosperity of civilizations have depended on the development of novel, more advanced materials. Today, the need for new materials is greater than ever, and the criteria for successful materials are becoming more stringent. On the one hand, growing awareness of the urgent need to reduce humans’ environmental footprint and reduce energy consumption is putting the focus on environmentally friendly synthetic methods which use as little energy as possible. On the other hand, low-cost, widely available materials are needed, especially in the biomedical field. Meeting both sets of requirements, however, is no easy task.

Nevertheless, examples of such materials are readily available. Materials synthesized by different organisms are produced from inexpensive raw materials, in low-energy and environmentally friendly processes [1,2]. Interestingly, the properties of many such biological materials are still unmatched by any engineered material [3,4,5,6]. However, biological materials are the result of an evolutionary process, usually highly specialized and therefore not necessarily optimized for all properties [3,7]. Moreover, they are formed from a limited number of constituents [8], which motivates and challenges materials scientists to improve on nature’s work.

Biomimetic synthesis is therefore receiving increasing attention, which is accompanied by advances in the understanding of natural synthetic pathways in various tissues. The term biomimetic is derived from the ancient Greek words *bios*, meaning life, and *mimesis*, meaning to imitate [9], and was coined by Otto H. Schmittt in 1969 [10]. In biomimetic synthesis, two aspects of natural processes can be mimicked: the specific properties or synthetic pathways of natural materials [11]. To date, biomimetic principles have been successfully applied in a wide range of fields, such as medicine [9], pharmaceuticals [12], robotics [13], bioelectronics [14], catalysis [15], energy [16], environmental protection [17], synthesis of nanomaterials [18], etc.

Among the various biological processes that inspire the development of novel materials, biomineralization, the formation of hard tissues by different organisms, takes a special place in the field [4,19,20]. In biomineralization, the organic matrix and/or soluble biomolecules exert strict control over the formation of the inorganic phase, including the control of its composition, morphology, and nucleation sites [1,2]. As a result, such tissues are organic–inorganic composites with complex, hierarchical structures (from the nano- to the micrometer scale), which differ in their properties from geological analogs of the same mineral counterparts [6]. Among the tissues that have attracted the most attention, due to their properties and functions they perform in organisms, are bone and nacre.

Bone is a family of composite materials whose basic structural unit is the collagen fibril mineralized with biological apatite [21,22,23,24]. Starting from the basic components, i.e., the mineral phase, collagen, non-collagenous proteins, proteoglycans, and water, to tissues and organs, nine different hierarchical levels can be distinguished [21]. Recent studies using the focused ion-beam electron microscope and serial surface view method revealed that the majority of the bone is composed of ordered arrays of mineralized collagen fibrils and macromolecules associated with them, while a minor part is composed of relatively disordered individual collagen fibrils with crystals located within and possibly between the fibrils [21]. Such structural organization results in a material with high strength and fracture toughness [5].

Nacre forms the inner layer of many shells and pearls. The nacre is structured from layers of submicrometer-sized aragonite tablets, often referred to as “bricks”, interconnected with organic components, the “mortar”. This “mortar” composed of β-chitin, diverse proteins and small organic molecules (polysaccharides, lipids, pigments, etc.) acts as a viscoelastic glue [6,25]. As a result, the strength of nacre is up to 3000 times higher than that of geological aragonite, which has no organic matter in its structure [2,8,26].

Although the interest in elucidating the underlying principles of biomineralization and their application in materials synthesis was initially motivated by the need to repair damaged hard tissue, it soon became apparent that this knowledge can be successfully applied in different fields, such as biosensing, drug delivery semiconductors, transportation, civil engineering, energy conversion and storage [6,20,27].

The papers presented in this Special Issue address several topics of interest for the design and application of biomimetic organic–inorganic composite materials in biomedicine.

The investigation of the role that individual amino acids (AAs) have in the precipitation process of calcium phosphates (CaPs) has been proposed as a way to both deepen the understanding of biomineralization [28] and to find a biomimetic route to improve the bioactivity of CaPs [29]. Despite extensive work in this area, the effect of individual AAs is still not fully understood due to conflicting results in the literature. Moreover, most of the investigations were performed on hydroxyapatite. Motivated by this, Mihelj Josipović et al. [30] investigated the influence of charged, polar, and non-polar AAs on calcium phosphate growth, initiated with octacalcium phosphate and dicalcium hydrogenphosphate dihydrate crystal seeds in metastable solution at physiological pH. It was shown that the influence of individual AAs on the rate of seeded growth and the properties of the solid phase formed depended on the type of seed applied.

Due to their similarity to bone structure and/or composition, mineralized, biodegradable, porous 3D scaffolds are receiving increasing attention in the field of bone tissue engineering [31,32]. Among these scaffolds, those based on collagen sponges (Col) are of special interest. Santhakuar et al. [33] described the preparation of collagen sponges coated with amorphous calcium phosphate (ACP) and low-crystalline apatite (Ap) and compared their bone regeneration capabilities in rat cranial defect model. Despite the fact that it was previously shown that the Col-ACP composite does induce apatite formation in SBF [34], it did not have beneficial effects on the healing of cranial effects, unlike the Col-Ap composite. The poor performance of the Col-ACP composite was attributed to several factors including an acidified environment as a consequence of postoperative inflammation and/or secretion of acid by osteoclasts resulting in increased solubility of ACP, the circulation of body fluid, type of the bone defect [33].

Another polymer of great interest for biomedical applications is hyaluronic acid [35]. This is a polysaccharide that constitutes an extracellular matrix in various parts of the human body, such as the joints, eyes, and skin [36,37]. In addition to its chemical and structural properties, its biocompatibility makes it a valuable capping, dispersing and templating agent for different biomedical applications [38]. Sikkema et al. [38] summarized the advances in the preparation, properties and applications of organic–inorganic hyaluronic-acid-based composite gels, films, coatings, scaffolds, biocements, bioceramics, bioglasses, and particles.

The physical and chemical properties of the surface of biomaterials and biomedical devices are among the key factors that determine the success of their application. To improve their biocompatibility, the biomaterial surfaces can be modified by various physical and chemical methods [39,40]. Arango-Satander [41] reviewed biomimetic topographic surface modifications of biomaterials aimed at reducing bacterial adhesion and improving cell attachment. The results of surface modifications inspired by the skin of various animals (shark, gecko), insects (dragonfly, planthopper) and plant surfaces (lotus, rose petals, floating fern, rice and taro leaves) were summarized and their potential as non-chemical alternatives to improve biomaterial performance was discussed.

These articles illustrate the wealth of research topics in the field of biomimetic organic–inorganic composites and contribute to the development of a framework for the rational design and synthesis of such materials.
